# Unconscious Affective Responses to Food

**DOI:** 10.1371/journal.pone.0160956

**Published:** 2016-08-08

**Authors:** Wataru Sato, Reiko Sawada, Yasutaka Kubota, Motomi Toichi, Tohru Fushiki

**Affiliations:** 1 Department of Neurodevelopmental Psychiatry, Habilitation and Rehabilitation, Graduate School of Medicine, Kyoto University, 53 Shogoin-Kawaharacho, Sakyo, Kyoto 606–8507, Japan; 2 Health and Medical Services Center, Shiga University, 1-1-1, Baba, Hikone, Shiga 522–8522, Japan; 3 Faculty of Human Health Science, Graduate School of Medicine, Kyoto University, 53 Shogoin-Kawaharacho, Sakyo-ku, Kyoto 606–8507, Japan; 4 The Organization for Promoting Neurodevelopmental Disorder Research, 40 Shogoin-Sannocho, Sakyo, Kyoto 606–8392, Japan; 5 Faculty of Agriculture, Ryukoku University, 1–5 Seta Oe-Cho Koya, Ohtsu, Shiga, 520–2194, Japan; University of Toyama, JAPAN

## Abstract

Affective or hedonic responses to food are crucial for humans, both advantageously (e.g., enhancing survival) and disadvantageously (e.g., promoting overeating and lifestyle-related disease). Although previous psychological studies have reported evidence of unconscious cognitive and behavioral processing related to food, it remains unknown whether affective reactions to food can be triggered unconsciously and its relationship with daily eating behaviors. We investigated these issues by using the subliminal affective priming paradigm. Photographs of food or corresponding mosaic images were presented in the peripheral visual field for 33 ms. Target photos of faces with emotionally neutral expressions were then presented, and participants rated their preferences for the faces. Eating behaviors were also assessed using questionnaires. The food images, relative to the mosaics, increased participants’ preference for subsequent target faces. Furthermore, the difference in the preference induced by food versus mosaic images was positively correlated with the tendency to engage in external eating. These results suggest that unconscious affective reactions are elicited by the sight of food and that these responses contribute to daily eating behaviors related to overeating.

## Introduction

Affective or hedonic responses (e.g., a feeling of pleasure [1]) to food have crucial consequences for humans of both an advantageous and disadvantageous nature. Throughout our long evolutionary history, because affects provide adaptive signals [2] and trigger multiple adaptive responses, including behavioral motivation, physiological preparation, and cognitive adjustment [3], they may have allowed our ancestors to seek and acquire valuable food in an environment scarce in food resources. However, because the current environment in many advanced nations has become one filled with high-calorie food and food advertisements, affective reactions to food may promote overeating and the risk of lifestyle-related diseases. Previous behavioral studies have confirmed that both the observation and consumption of food elicit positive affective responses [4,5], which in turn stimulate food intake [6,7]. In line with these findings, functional neuroimaging studies revealed that the observation and consumption of food activate brain regions related to affective processing, such as the amygdala and nucleus accumbens (e.g., [8]; for a review, see [9]).

Previous behavioral studies have shown that information related to food can be processed rapidly, even without conscious awareness [10–12], a finding that is consistent with the critical importance of food for survival. For example, one study reported that subliminally presented images of fast food augmented the speed of cognitive judgments [10]. Another study reported that the subliminal presentation of food images enhanced the behavioral response of acquiring a subsequent food reward. These data suggest that both cognitive and behavioral responses to food can be unconsciously triggered [12].

However, whether affective responses to food can be unconsciously elicited remains unknown. Although it is possible to speculate that the cognitive and behavioral responses to food found in previous research could be characterized as affective in nature, these responses may have been elicited without the involvement of affects. Subjective affective experience provides a direct source of evidence for affective responses, but no study has investigated subjective affective responses to subliminally presented stimuli related to food. In other areas of the literature, several behavioral studies have used the subliminal affective priming paradigm and found that non-food affective stimuli, such as facial expressions of emotion, elicited unconscious affective responses (e.g., [13]; for a review, see [14]). In a typical use of this paradigm, a facial expression depicting a positive or neutral emotion is briefly flashed as a prime, and a neutral target (e.g., a neutral face) is then presented. Participants are asked to evaluate the target in terms of preference. Previous studies have reported that evaluations of the target are positively biased when unconsciously perceived positive stimuli appear as primes in comparison with neutral stimuli used as primes. This effect is regarded as evidence that unconscious affective reactions are elicited and then spill over into the affective evaluations of unrelated targets [13]. This indirect priming procedure appears to detect unconscious affective reactions more sensitively than do direct evaluations of subjective experiences [15]. Functional neuroimaging studies have also revealed that the subliminal presentation of non-food affective stimuli, such as emotional facial expressions, activates affect-related brain regions such as the amygdala and nucleus accumbens [16]. Based on these data demonstrating unconscious affective processing for non-food stimuli, together with the above evidence for the affective and unconscious processing of food-related stimuli, we hypothesized that affective reactions to food would also be triggered unconsciously.

Furthermore, we expected that unconscious affective responses to food would influence eating habits and tendencies in daily life. Previous behavioral studies have shown that food related tendencies can be measured with good reliability and validity using self-report questionnaires such as the Dutch Eating Behavior Questionnaire (DEBQ) [17]. The DEBQ measures tendencies related to external eating, restrained eating, and emotional eating, all of which may contribute to overeating [17]. Among these tendencies, several previous studies have reported that the tendency to engage in external eating, defined as a tendency to eat in response to external cues, such as the sight or smell of food, modulates automatic processes related to food, such as the allocation of attention to food [18–20]. Furthermore, a neuroimaging study found that the external eating tendency modulated functional connectivity between the amygdala and nucleus accumbens during the observation of food images [21]. Based on these data, we hypothesized that unconscious affective responses to food may be related to the external eating tendency.

To test these hypotheses, we investigated unconscious, as well as conscious, affective responses to food and non-food stimuli and the relationships of these responses to eating behaviors. To investigate unconscious affective responses, we used the subliminal affective priming paradigm [22]. Food images and their corresponding mosaics were presented as primes for 33 ms in the peripheral visual field (i.e., outside participants’ attentional focus), followed by a mask. Neutral faces were subsequently presented, and participants rated their preferences for the target faces. To investigate the generalizability of the effects across various types of food, we presented two food types: fast food items and items from a traditional Japanese diet. To investigate conscious affective responses, we presented the food and mosaic stimuli to participants supraliminally, and participants then explicitly rated these stimuli in terms of preference. We did not use the priming procedure under the supraliminal condition for two reasons: first, we wanted to directly assess affective reactions to the stimuli; and second, a previous study showed that conscious evaluations cancel the priming effect for supraliminally presented affective stimuli [13]. We tested the effect of stimulus type (food versus mosaic) based on preference ratings for subsequent faces under the subliminal condition and for the stimuli *per se* under the supraliminal condition. We also assessed eating tendencies using the DEBQ, as well as body mass index (BMI) and the participants’ level of hunger, all of which are potentially related to food processing [23,24]. We then tested the relationships between each of these variables and the unconscious and conscious preference ratings for food-related stimuli. We also investigated performance in a forced-choice recognition task with subliminally presented food stimuli to confirm the processing without conscious awareness.

## Materials and Methods

### Ethics Statement

This study was approved by the Ethics Committee of Graduate School of Medicine, Kyoto University. The experiment was conducted at Kyoto University in accordance with the approved guidelines. All participants gave written informed consent after being provided with an explanation of the experimental procedure.

### Participants

The study included 34 healthy Japanese volunteers (16 females and 18 males; mean ± *SD* age, 23.3 ± 4.5 years; mean ± *SD* BMI, 21.6 ± 3.4). Six additional volunteers participated, but their data were not analyzed for the following reasons: (1) three participants reported that they had observed food images in the subliminal condition and correctly reported multiple food categories during the debriefing process; and (2) three participants reported a level of hunger below the neutral state. All participants were right-handed, as assessed by the Edinburgh Handedness Inventory [25], and had normal or corrected-to-normal visual acuity. All were naïve with respect to the purpose of the experiment and fasted for at least 3 h before the experiment.

### Experimental design

The experiment was constructed as a within-subjects three-factorial design, with stimulus type (food/mosaic), food type (fast food/Japanese diet), and visual field (left/right) as the factors. Dependent variables were preference ratings for faces and food/mosaic stimuli under the subliminal and supraliminal presentation conditions, respectively.

### Stimuli

**Fig 1:**
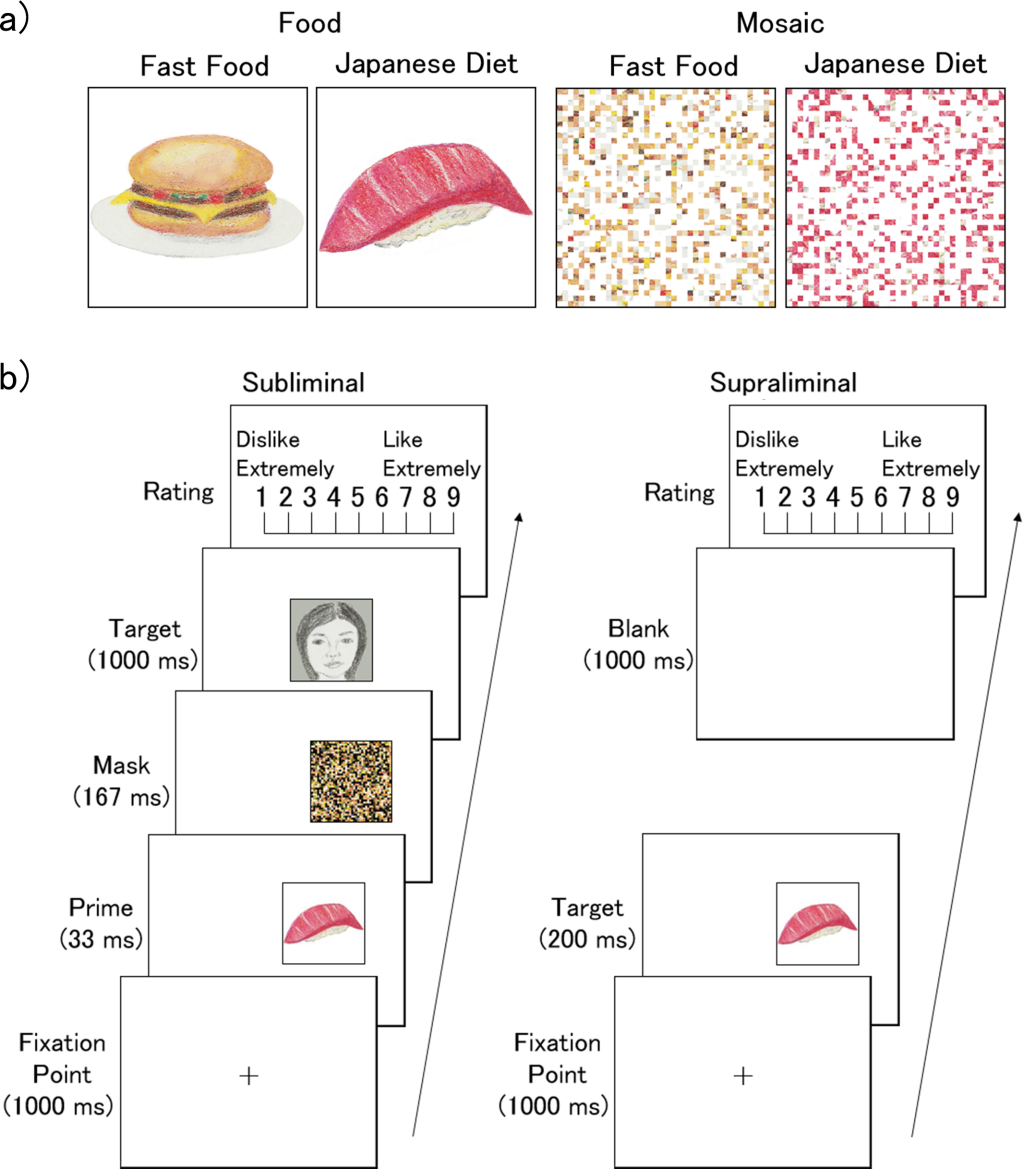
**Illustrations of food and mosaic stimuli (a) and the trial sequences of the subliminal and supraliminal presentation conditions (b).** In the actual experiment, photographic stimuli were used.

Food stimuli were color photographs of fast food (three images within each of the four following subcategories: hamburgers, fried chicken, pizza, and doughnuts) and traditional Japanese diet (three images within each of the four following subcategories: sushi, roast fish, Japanese mixed rice, and noodles). The photographs were selected from images appearing on web sites and were cropped using PhotoShop CS6 (Adobe). The size of the stimuli was 7.0° vertically × 7.0° horizontally. Illustrations of the stimuli are shown in Fig 1 (also see S1 Fig for more examples).

The mosaic stimuli were constructed from the food stimuli using MATLAB 6.5 (MathWorks). First, all food stimuli were divided into small squares (40 vertical × 40 horizontal) and reordered using a randomization algorithm. This rearrangement rendered each image unrecognizable as food. A mask stimulus was also prepared by creating a mosaic pattern made up of fragments of food images not used in the experiment.

Face images were prepared as target stimuli for the preference-rating task under the subliminal presentation condition. Faces were grayscale photographs depicting full-face, emotionally neutral expressions displayed by 48 (24 female and 24 male) Japanese models. To ensure that the target stimuli were affectively neutral, we initially prepared 65 face images and presented them to 14 participants (none of whom took part in the experiment itself). These participants provided preference ratings for these stimuli using a 5-point scale, and 48 models rated as relatively neutral were chosen as the target stimuli. The target stimuli were randomly assigned to the experimental conditions (stimulus type/food type/visual field) and appeared once in each of the first and second blocks. The size of the stimuli was 7.0° vertically × 7.0° horizontally.

#### Apparatus

Stimulus presentation was controlled by Presentation 14.9 (Neurobehavioral Systems) implemented on a Windows computer (HP Z200 SFF, Hewlett-Packard Company). The stimuli were presented on a 19-inch CRT monitor (HM903D-A, Iiyama) with a refresh rate of 150 Hz and a resolution of 1024 × 768 pixels. The refresh rate was confirmed by a high-speed camera (EXILIM FH100, Casio) with a temporal resolution of 1000 frames/s.

#### Questionnaires

We used the Japanese version of the DEBQ [17,26] to measure eating tendencies. The questionnaire included 33 items that assessed three tendencies related to overeating: restrained eating (10 items, e.g., “Do you deliberately eat less in order to not become heavier?”), emotional eating (13 items, e.g., “Do you have the desire to eat when you are irritated?”), and external eating (10 items, e.g., “If you see or smell something delicious, do you have a desire to eat it?”). Each item was scored on a scale from 1 (seldom) to 5 (very often). The reliability and validity of the questionnaire were confirmed against the original [17] and Japanese [26] versions.

We also assessed participants’ BMI and their level of hunger on a 5-point scale from 1 (not at all) to 5 (extremely). Participants used an electric calculator to compute their BMI based on an equation that was provided to them.

#### Procedure

The experiments were conducted individually in a soundproofed room (Science Cabin, Takahashi Kensetsu). Upon arrival, participants were explained that they would evaluate various types of stimuli in terms of preference during the experimental tasks. Participants were first asked to fill out questionnaires pertaining to level of hunger. They then were seated 0.57 m from the monitor and performed the experimental tasks.

Within each of the subliminal and supraliminal presentation conditions, a total of 96 trials involving preference judgments (12 fast food, 12 Japanese diet, 12 fast food mosaic, and 12 Japanese diet mosaic images shown in each of the left and right visual fields) were performed (i.e., for a total of 192 trials). Participants completed these trials in two blocks of 48. Each block contained an equal number of trials for each of the stimulus type/food type/visual field conditions. The order of conditions was randomized within each block. A short break occurred after each block, and a longer break occurred after the completion of the subliminal condition. Participants initially participated in five practice trials to become familiar with the procedure in each presentation condition.

In each trial within the subliminal presentation condition (Fig 1; also see S2 Fig for more illustrations), a cross was initially presented for 1000 ms as a fixation point at the center of the visual field. A prime food or mosaic image was then presented for 33 ms in the left or the right visual field (the inside edge was 3.5° peripheral to the center); this was immediately followed by the presentation of a mask stimulus in the same location for 167 ms. The exposure duration of the prime and mask stimuli were determined based on data from previous studies using subliminal presentation [27] and the results of our own preliminary studies. The target face was then immediately presented in the central visual field for 1000 ms. Finally, the rating display was presented until the participant entered a response. Participants were instructed to maintain their gaze throughout the trial at the location of the fixation cross. The participants’ task was to rate their preference for the target faces using a 9-point scale ranging from “dislike extremely” to “like extremely” [28]. They were asked to respond by pressing keys with their right index finger.

In each trial within the supraliminal presentation condition (Fig 1; also see S2 Fig for more illustrations), after a cross appeared for 1000 ms as a fixation point at the center of the visual field, a target food or mosaic image was presented for 200 ms in the left or the right visual field (the inside edge was 3.5° peripheral to the center). After the presentation of a blank screen of 1000 ms, the rating display was presented until the participant entered a response. Participants were instructed to maintain their gaze throughout the trial at the location where the fixation cross had appeared. Their task was to rate their preferences for the target food/mosaic images using the same 9-point scale as in the subliminal presentation condition.

As an objective approach to measuring the subliminal effect of the prime stimuli, a forced-choice discrimination task was administered after the preference ratings were complete, as in previous studies [13,22]. A total of 48 trials were conducted using food stimuli.

In each trial, a food stimulus followed by a mask was presented in the same manner as under the subliminal presentation condition. Then, the two food stimuli, one of which had been presented as the prime, were presented in the upper and lower visual fields. The two stimuli were in the same food subcategory, and the participants indicated which food had been presented before. This task was based on the assumption that participants who had acquired a conscious awareness of food stimuli would be able to select the stimuli based on low-level visual information.

After completing the above procedure, participants were asked to fill out the questionnaire pertaining to DEBQ and BMI. An interview was subsequently conducted and participants were asked whether they had consciously perceived the primes during the subliminal presentation condition. Debriefing was then conducted. After explaining the purpose of the experiment, we requested the participants’ permission to analyze their data under the subliminal presentation condition; all of the participants consented to this request.

#### Data analysis

Data were analyzed using SPSS 16.0J software (SPSS Japan). The preference rating data were analyzed separately for each presentation condition using a three-way repeated-measures analysis of variance (ANOVA) with stimulus type (food/mosaic), food type (fast food/Japanese food), and visual field (left/right) as within-participant factors. Because our preliminary analyses showed that the sex and age of the participants had no significant effects on the results, these factors were disregarded.

To investigate the relationship between affective responses to food and eating tendencies, differences between the preference ratings for food versus mosaic conditions were calculated as a score for each participant’s food preference under each presentation condition. We then calculated Pearson’s product–moment correlations between food preference scores and DBEQ external eating scores. Based on our interest, the significance of the correlation coefficients was evaluated using *t*-tests (two-tailed). For descriptive purposes, we also tested other correlations using *t*-tests (two-tailed), although we had made no specific predictions regarding these.

The forced-choice discrimination data were analyzed using one-sample *t-*tests.

Results were considered statistically significant at *p* < 0.05.

## Results

### Preference evaluation

**Fig 2:**
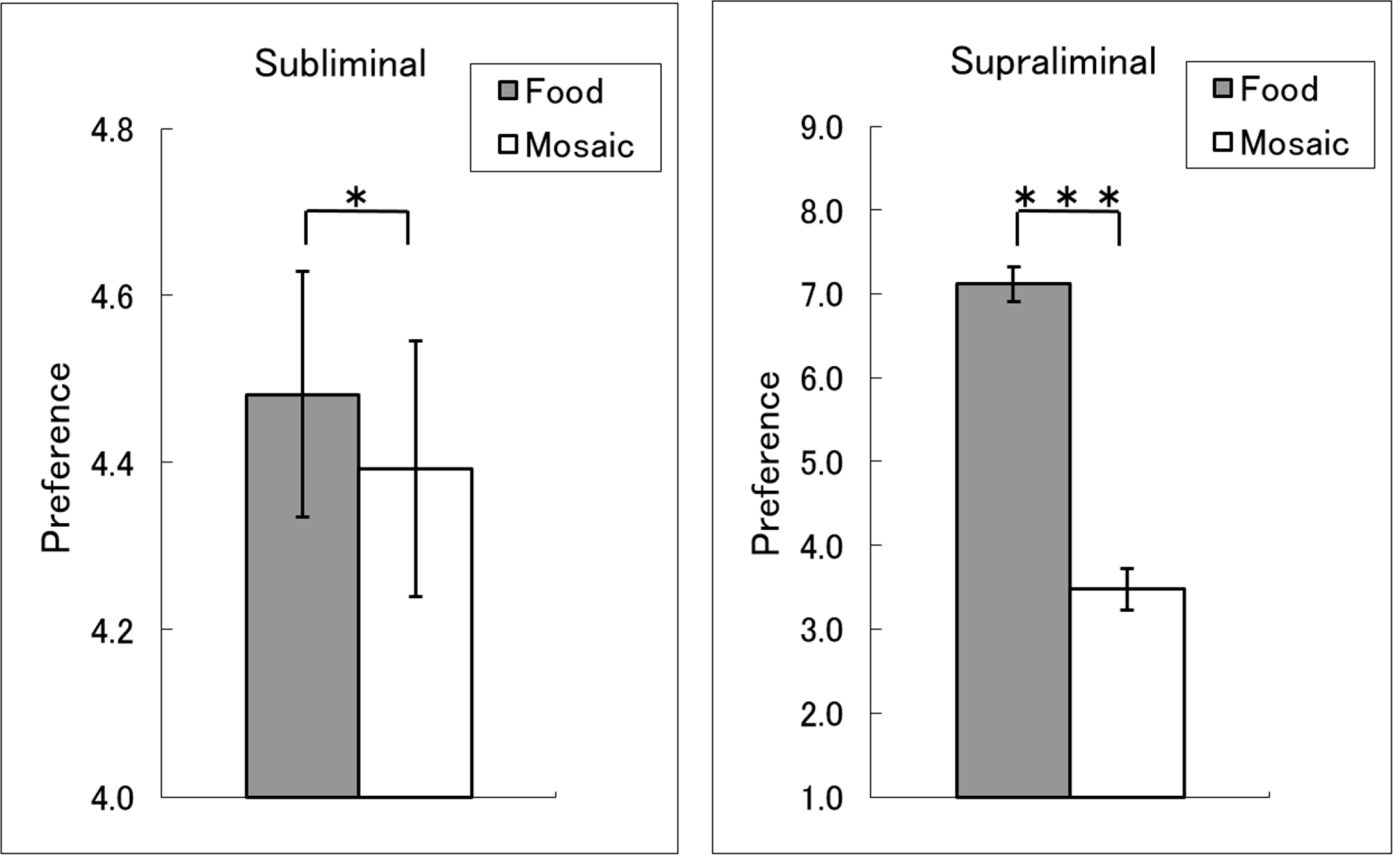
**Mean (with *SE*) preference ratings under the subliminal (left) and supraliminal (right) presentation conditions.** The ratings are for faces and food/mosaic stimuli under the subliminal and supraliminal presentation conditions, respectively. Asterisks indicate significant effects of stimulus type (*** *p* < 0.001; * *p* < 0.05).

To evaluate food-triggered preferences in the subliminal condition (Table 1; Fig 2; S3 Fig), a three-way ANOVA was conducted with stimulus type, food type, and visual field as factors. This analysis revealed a significant main effect of stimulus type (*F*(1,33) = 6.46, *p* < 0.05, *η*_*p*_^2^ = 0.16), indicating that preference ratings for faces primed by food images were higher than those for faces primed by mosaics. There were no other significant main effects or interactions (*F*(1,33) < 3.44, *p* > 0.05).

**Table 1:** Mean (with *SE*) preference ratings.

Presentation	Visual Field	Food		Mosaic	
		Fast	Japanese	Fast	Japanese
Subliminal^a^	Left	4.53	4.47	4.56	4.37
		(0.16)	(0.15)	(0.17)	(0.15)
	Right	4.48	4.45	4.35	4.29
		(0.15)	(0.13)	(0.14)	(0.16)
Supraliminal^b^	Left	7.13	7.01	3.48	3.37
		(0.21)	(0.19)	(0.25)	(0.24)
	Right	7.25	7.06	3.65	3.41
		(0.21)	(0.21)	(0.25)	(0.23)

^a^ Preference ratings for faces with food and mosaic primes.

^b^ Preference ratings for food and mosaic stimuli.

Preference ratings in the supraliminal condition (Table 1; Fig 2; S3 Fig) showed a similar pattern. A three-way ANOVA revealed a significant main effect of stimulus type (*F*(1,33) = 178.05, *p* < 0.001, *η*_*p*_^2^ = 0.84), indicating that preference ratings for food images were higher than those for mosaics. The main effect of visual field was also significant (*F*(1,33) = 11.30, *p* < 0.01, *η*_*p*_^2^ = 0.25), indicating that preference ratings were higher for stimuli presented in the right visual field than for stimuli appearing in the left visual field. There were no other significant main effects or interactions (*F*(1,33) < 3.27, *p* > 0.05).

#### Relationship between the preference for food stimuli and eating tendencies

**Fig 3:**
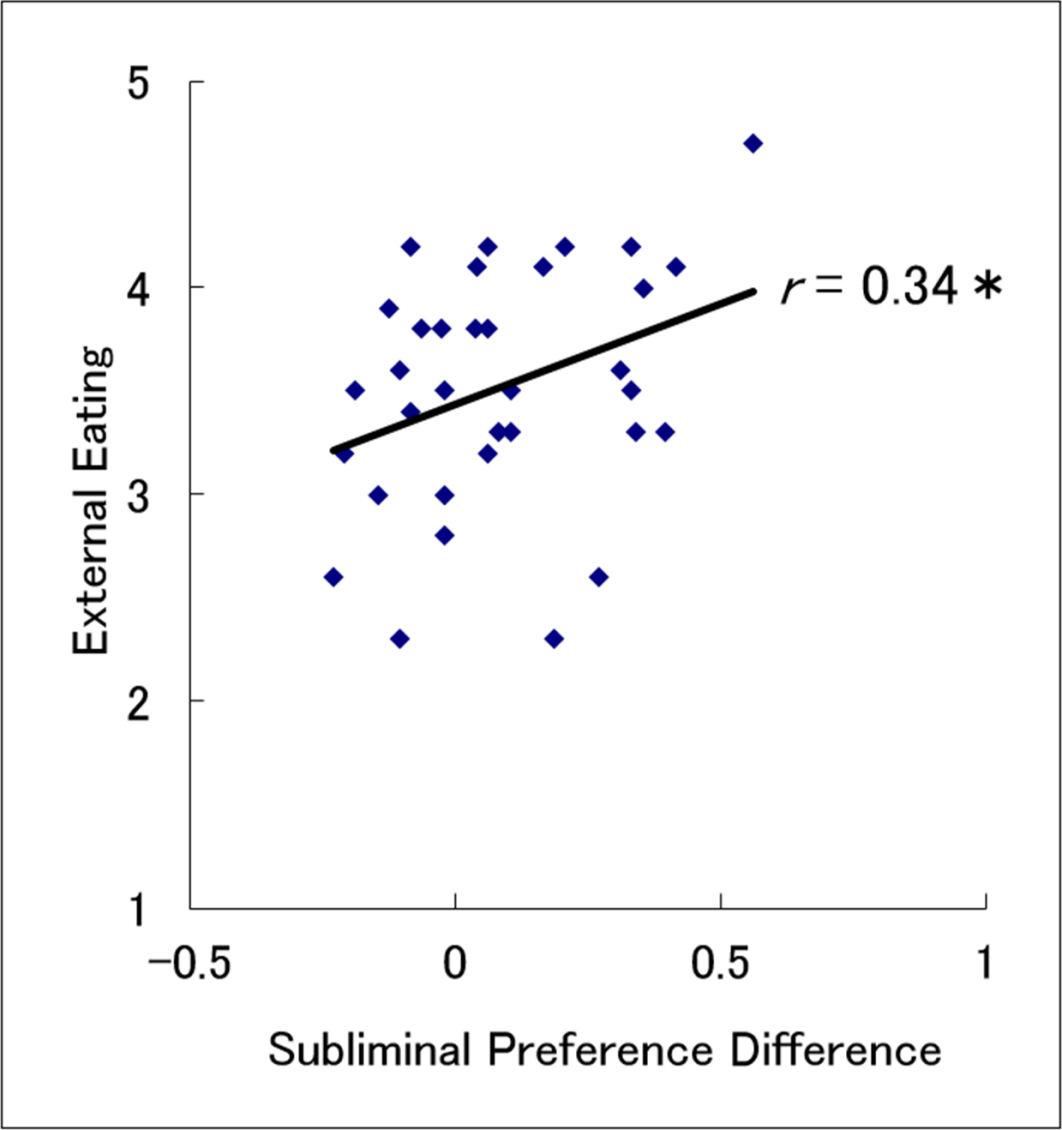
A scatter plot with regression lines of food preference scores under the subliminal condition as a function of external eating tendency.

Table 2 presents the means (± *SE*) of food preference scores (differences in preference ratings between the food and mosaic conditions) under the subliminal and supraliminal presentation conditions, along with DEBQ tendency scores related to restrained, emotional, and external eating, participants’ BMI values, and ratings related to the participants’ level of hunger. Correlations were analyzed to investigate relationships between food preference scores under each presentation condition and the other variables (Table 2). Consistent with our hypothesis, we found a significant positive correlation between food preference scores under the subliminal condition and scores related to the external eating tendency (*r* = 0.34, *p* < 0.05; Fig 3). When we explored food preference scores under the supraliminal condition, the correlation with external eating tendency scores did not reach significance (*r* = 0.28, *p* > 0.1). We also explored correlations between food preference scores and other DEBQ tendencies (restrained eating and emotional eating), degree of hunger, and BMI. We found that only a negative correlation between food preference scores in the subliminal condition and the restrained eating tendency scores reached significance (*r* = -0.36, *p* < 0.05).

**Table 2:** Mean (with *SE*) data and their correlations.

Data		*M*	*SE*	Correlation					
				1	2	3	4	5	6
1	Subliminal Preference^a^	0.09	0.03						
2	Supraliminal Preference^b^	3.64	0.27	0.02					
3	Restrained Eating	2.58	0.13	-0.36*	0.07				
4	Emotional Eating	2.61	0.17	0.05	0.20	0.10			
5	External Eating	3.52	0.10	0.34*	0.27	-0.30	0.27		
6	Body Mass Index	21.60	0.59	-0.19	0.27	0.15	0.12	-0.03	
7	Hunger Degree	4.00	0.10	-0.01	0.21	-0.24	0.01	0.20	-0.27

* p < 0.05.

^a^ Differences in preference ratings for faces between food and mosaic prime conditions.

^b^ Differences in preference ratings for food and mosaic stimuli.

### Forced choice discrimination

The mean ± *SE* percentage of correct recognition responses was 51.0　± 1.7%. One-sample t-tests were performed to identify differences that exceeded the level of chance, and the results showed that the percentage of correct discrimination did not significantly differ from chance in either experiment (*t*(34) = 0.62, *p* > 0.1). These results serve as an objective indication that the primes had been presented subliminally under the present experimental conditions [29]. The debriefing interview further confirmed that none of the participants whose data were analyzed were consciously aware of the primes.

## Discussion

Our results from the supraliminal condition showed that the observation of food images induced higher preference ratings than the observation of mosaics. This result confirms the ample existing evidence that the observation of food induces affectively positive reactions [6,7].

More important, our results from the subliminal condition revealed that unconsciously perceived food primes heightened participants’ preferences for subsequent target faces relative to the mosaic primes. This result corroborates previous behavioral and neuroimaging studies reporting unconscious affective reactions to non-food images such as emotional facial expressions [13,16]. It is also consistent with previous findings showing that subliminally presented food images triggered multiple cognitive and behavioral responses [10–12]. However, to date, no study has reported evidence for unconscious affective reactions to food. To our knowledge, this is the first evidence indicating that unconscious affective reactions are elicited by food-related stimuli.

Furthermore, the results of our correlational analyses demonstrated that the unconscious preference for food stimuli over mosaics was positively related to the external eating tendency. This result is in line with previous behavioral findings showing that the automatic processing of food stimuli is positively associated with the external eating tendency [18–20] and with neuroimaging results indicating that functional connectivity among affect-related brain regions during the observation of food is positively modulated by the external eating tendency [21]. Our results extend the existing literature into the domain of unconscious affective processing related to food. Because the external eating tendency score indicates a tendency for food intake to be promoted by external stimuli such as the sight of food, our results suggest that affective reactions without conscious awareness play an important role in triggering such externally driven eating habits in daily life. Our results also align with those of a path analysis indicating that the positive relationship between the external eating tendency and overeating is mediated by food cravings [30]; craving is reportedly related to amygdala activation, suggesting it has an affective basis [31]. Because the relationship between food preference scores and external eating tendency scores was more pronounced under the subliminal condition than it was under the supraliminal condition, we speculate that eating behaviors in daily life may be largely affected by affective responses that are unconscious rather than conscious and reflective.

Our exploration of other eating tendencies revealed that unconscious food preference scores during hungry states were negatively correlated with the restrained eating tendency. Some recent studies have suggested that the restrained eating tendency as measured by the DEBQ consists of items addressing actual behavioral restraint and items related to intention to exercise restraint [32] and that it reflects the successful restriction of eating, primarily, among non-obese [30]. Because most of the present participants were in the normal weight range, their restrained eating scores may have reflected successful behavioral restriction. The results may suggest that individuals with weak unconscious affective responses to food are more successful than others in restricting their food intake.

Our results also showed that the food and mosaic stimuli presented in the right visual field, which involved processing by the left hemisphere, elicited a more pronounced preference than those presented in the left visual field, under the supraliminal presentation conditions. These results may be in line with some previous behavioral findings among normal and brain-damaged participants showing that the left hemisphere is dominant for positive affective processing [33]. A previous study also reported that stimulation of the left hemisphere using unilateral forced nostril breathing enhanced affective processing [34]. However, it should be noted that this effect was not specific to food; the left hemisphere appears generally dominant with respect to conscious positive affective responses.

Our results have several practical implications. First, the results pertaining to unconscious affective responses to food and the relationship of these responses to eating tendencies indicate that unconsciously elicited affects play an important role in daily eating behaviors. Because previous studies have indicated that humans consciously perceive stimuli only in very restricted areas available for focused attention [35] and that unconscious affective reactions are influenced very little by conscious reflection [13], unconscious affective responses to food may be widespread and uncontrollable. Affective responses to food may have critical consequences such as triggering overeating, which in turn increases the risk of developing diseases such as hypertension and type-2 diabetes [36]. We recommend that people who want to control their eating behaviors recognize the power of their unconscious affective responses and carefully control their environment, given that the mere sight of food within the peripheral visual field, unconsciously perceived, has the potential to trigger affects and promote eating behaviors.

Second, our results suggest that food can be used to modulate affects in the absence of conscious awareness. Our target stimuli in the subliminal presentation condition were face images; our results suggest that food-related stimuli that are irrelevant and unconsciously perceived may enhance preferences related to people. Hence, food in the environment during meetings, on romantic dates, or in various other social interaction contexts may increase positive attitudes toward other people, all in the absence of the conscious recognition of such influence.

It is worth noting some limitations of the present study, along with suggestions for future directions for this line of research. First, we used mosaic images to control for low-level visual features (e.g., overall luminance and color). Because the mosaics lacked multiple kinds of information associated with food, the specific information related to affective processing of food remains unclear. Future studies using different control stimuli, such as non-food objects, may be promising to further investigate the unconscious affective responses to food that were found in our study.

Second, we administered the DEBQ questionnaires immediately after the experiments, as has been done in several previous studies, and this procedure may not have been ideal for the measurement of daily eating behaviors. It is possible that the observations and evaluations of food images altered the transient food processing of some participants which, in turn, had a confounding effect on the eating behavior ratings. The administration of questionnaires under calm conditions outside the context of the experiment would be preferable for rigorously examining the daily eating behaviors in future studies.

Third, we did not find clear evidence that unconscious affective reactions to food were related to participants’ BMI and hunger levels. However, it should be noted that our data lacked sufficient variance with respect to these variables due to the lack of recruitment of obese participants and the fact that participants were only instructed to fast for a relatively short time prior to the experiment (i.e., 3 h). Previous studies have suggested that these factors may modify affective responses to food, showing, specifically, that obese participants showed heightened food preference scores relative to their lean counterparts [24] and that hunger states induced by a 9-h fast modulated attention to food [23]. Future studies investigating participants with high BMI or longer fasting times may reveal the modulatory effects of these factors on unconscious affective responses to food, which could provide insights into their underlying mechanisms with implications for real-life eating.

Finally, although we did not find clear effects for food type, this result should not be regarded as evidence that food type has no effect on unconscious affective reactions. We only presented images of a few subcategories of fast food and Japanese diet, all of which were familiar and palatable to Japanese participants. However, it is reasonable to expect that foods that are part of Japanese diet may have different effects on participants from other cultures. Furthermore, it may also be possible that highly palatable versus unpalatable food subcategories would elicit different unconscious affective reactions. It would be an interesting matter for future research to investigate the effect of food types and subcategories on unconscious affective reactions.

In summary, our results showed that subliminally presented food images increased preference ratings for subsequent target faces relative to mosaic images. Furthermore, preference scores in the subliminal condition were positively correlated with external eating tendencies. These results suggest that unconscious affective reactions are elicited by the sight of food, which in turn contribute to daily behaviors related to overeating.

## Supporting Information

S1 Dataset Datasets of participants’ background information, preference ratings, forced choice recognition rates, and eating behavior scores. (XLSX)

S1 Fig Illustrations of subcategories of food stimuli. In the actual experiment, photographic stimuli were used.
(TIF)

S2 Fig Illustrations of the trial sequences of the subliminal food, subliminal mosaic, supraliminal food, and supraliminal mosaic conditions. (TIF)

S3 Fig **Mean (with 95% confidence interval) differences in preference ratings between food versus mosaic conditions under the subliminal (left) and supraliminal (right) presentation conditions.** The ratings are for faces and food/mosaic stimuli under the subliminal and supraliminal presentation conditions, respectively.
(TIF)
