# Supplementary figures and images for: Unconscious Affective Responses to Food
Source: PLoS One. 2016 Aug 8;11(8):e0160956. doi: 10.1371/journal.pone.0160956 (PMC4976966; doi:10.1371/journal.pone.0160956)

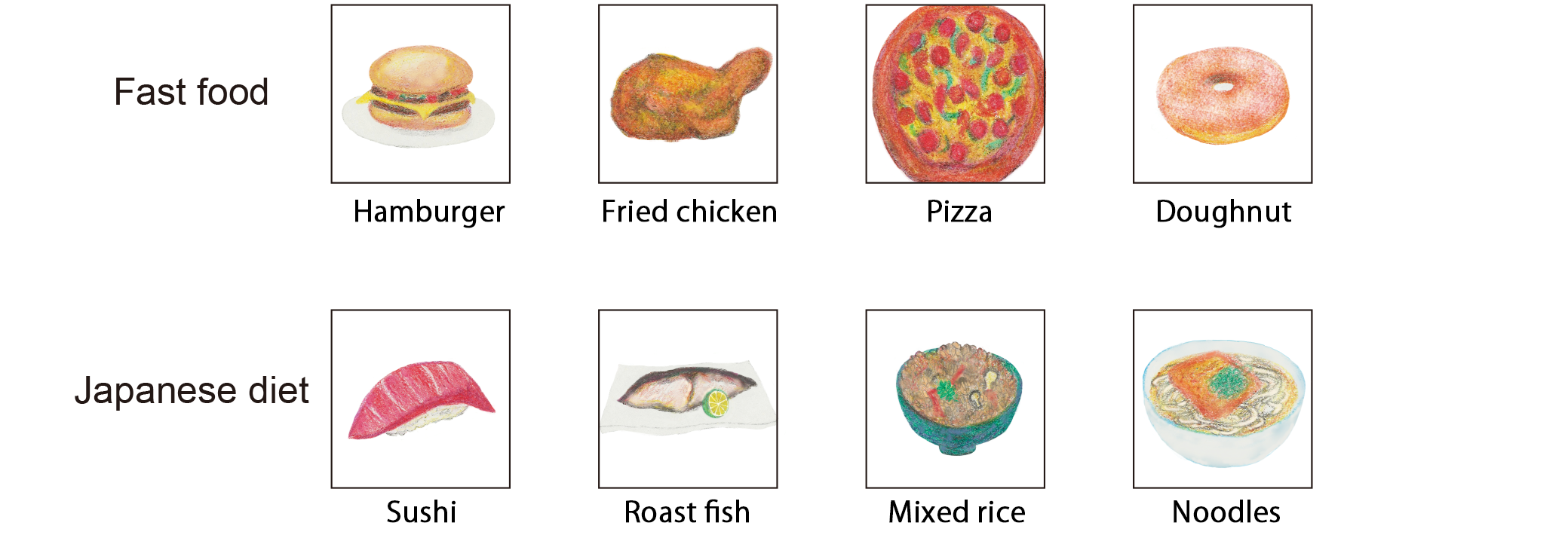

Supplement: S1 Fig — In the actual experiment, photographic stimuli were used. (TIF) [file pone.0160956.s002.tif]

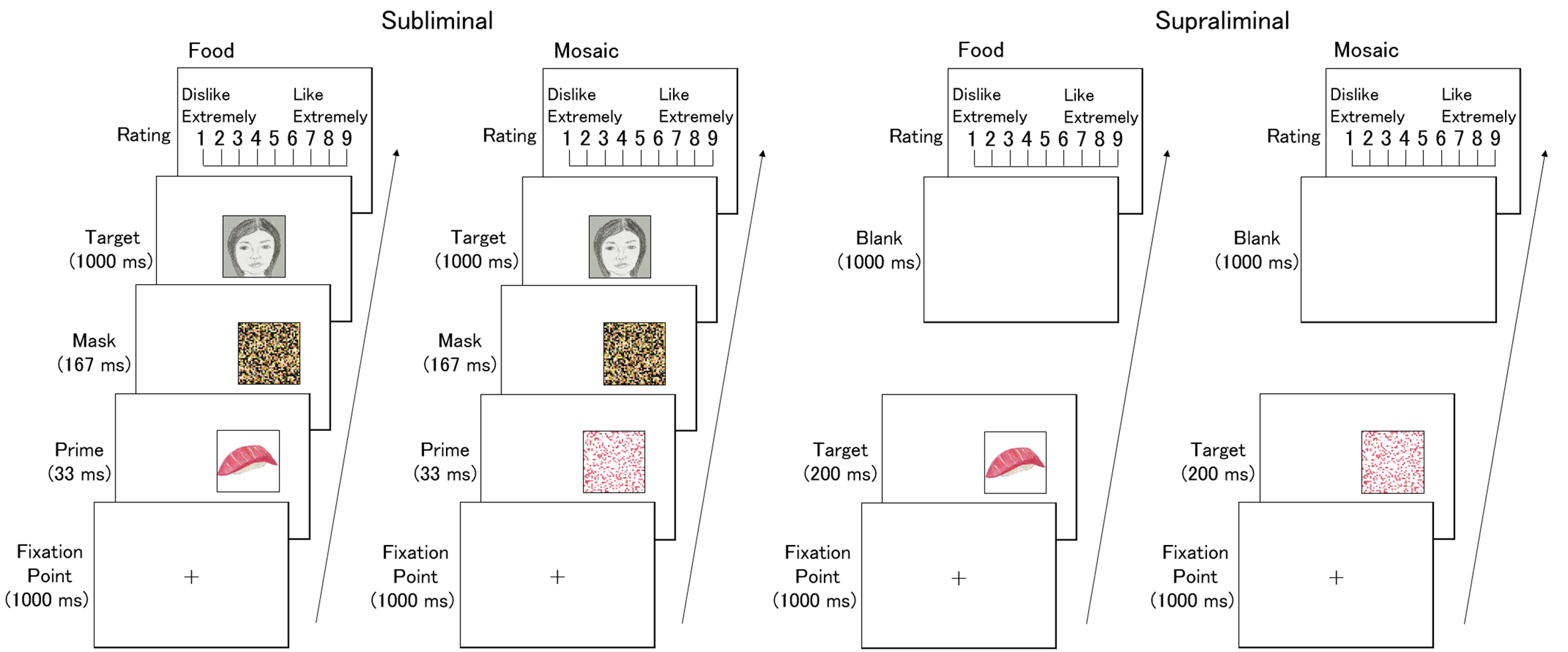

Supplement: S2 Fig — (TIF) [file pone.0160956.s003.tif]

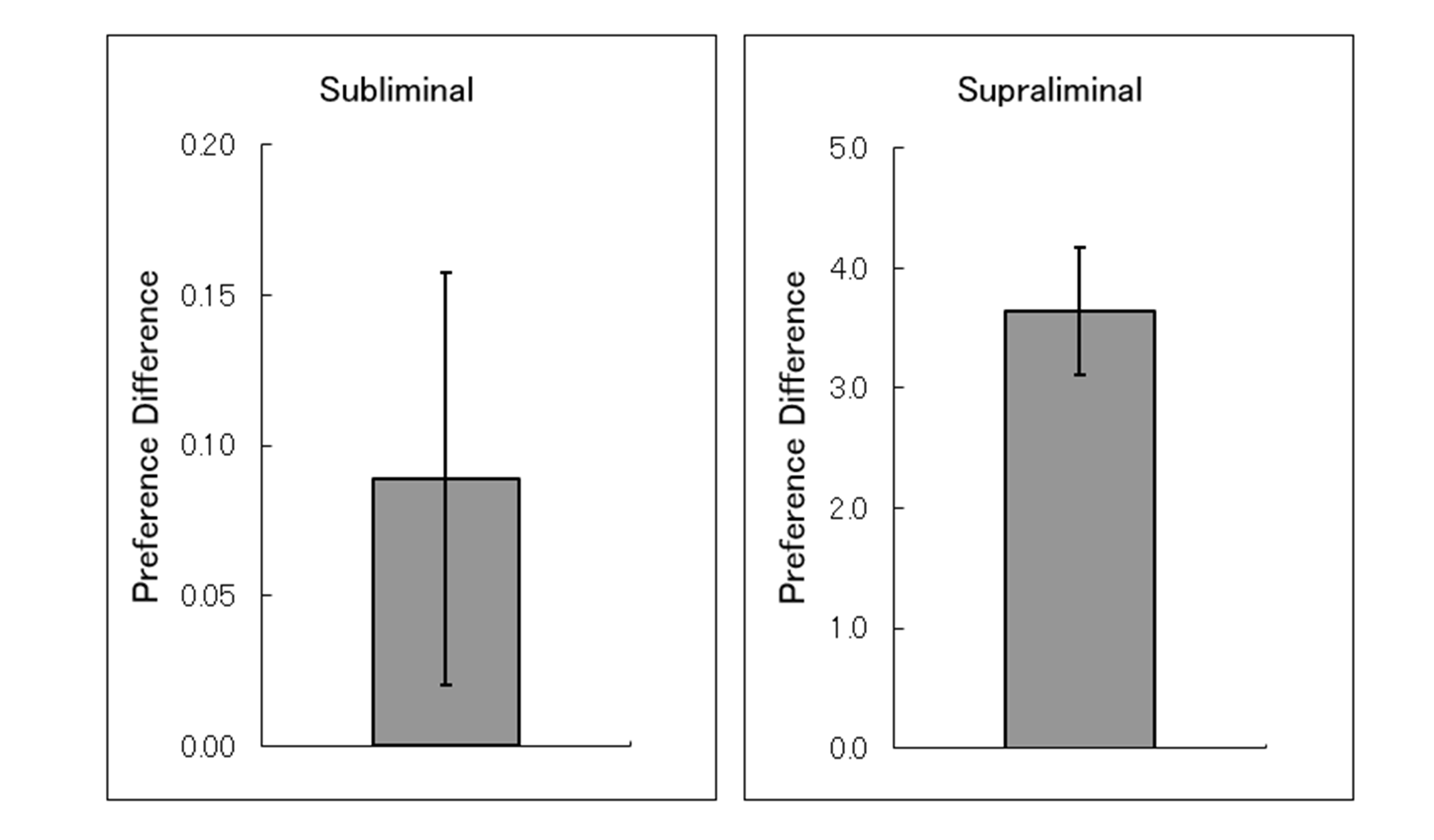

Supplement: S3 Fig — Mean (with 95% confidence interval) differences in preference ratings between food versus mosaic conditions under the subliminal (left) and supraliminal (right) presentation conditions. The ratings are for faces and food/mosaic stimuli under the subliminal and supraliminal presentation conditions, respectively. (TIF) [file pone.0160956.s004.tif]
